# Regular Meeting of the Fulton County Medical Society, February 3, 1916

**Published:** 1916-02

**Authors:** Allen H. Bunce


### REGULAR MEETING OF THE FULTON COUNTY MEDICAL SOCIETY, FEBRUARY 3, 1916.

(Reported by Allen H. Bunce, M. D.)

Dr. Roy Blosser exhibited a new Abdominal Supporter which he has found very beneficial in his work. In cases of gastroptosis and splanchnoptosis he has used it with gratifying results. In thirty-five cases reported he states that patients averaged a gain of ten pounds each.

Dr. T. C. Davison read a paper on "Intravenous Treatment of Acute Rheumatism." It appears elsewhere in the Journal.

In discussing Dr. Davison's paper, Dr. Hugh Lokey stated that he had given Sodium Salicylate per rectum with very satisfactory results. He uses 40 to 60 grains of the true Salicylate of Soda from Oil of Wintergreen dissolved in 4 ounces of water. It is injected with a colon tube with the patient lying on the abdomen and retained over night. Previous to the injection a cleansing enema is given. In cases of rheumatic irritis he has found this extremely beneficial where patients did not tolerate the Salicylate taken by the mouth.

Dr. C. C. Aven highly commended the work of Dr. Davison in the intravenous medication for acute articular rheumatism. He reported using this method with satisfactory results where other methods failed. In his work he uses only the true Sodium Salicylate.

Dr. L. M. Gaines read a paper on "Epidemic Cerebro-Spinal Meningitis, Developing 22 Hours after Abdominal Operation."

Dr. E. C. Davis said that he wished to commend the work reported since only efficient and accurate work in this case saved the patient. He said that it emphasized the importance of examining patients carefully and finding the cause of complications which may arise.

Dr. A. H. Lindorme read a paper: "Report of an Interesting Case of Inherited Syphilis." It appears elsewhere in the Journal.

Dr. M. T Davis said, "Dr. Lindorme's paper is both instructive and timely, since any discussion of Syphilis to-day, must interest not only the Genito-Urinary specialist, but the internist and indeed all others engaged in the practice of Medicine, since Syphilis spares no tissue in the human body.

"Acquired syphilis in childhood is not common but congenital syphilis confronts us everywhere. To diagnose it is the important point, but the history of the case often leaves us in the dark, the mother having no knowledge of any infection or else denying it. The child itself may have had no secondary manifestations or else they may have been so mild as to have been overlooked. And yet in from ten to twenty-five years, this same patient will develop symptoms of tertiary lues, the so-called hereditary syphilis.

"We should be on the alert in the diagnosis of congenital syphilis, and it is important to examine other children of the same family. For instance, a little patient is brought to us on account of headache and fatigue. The mother knows nothing of syphilis, doesn't even know what it is. The child shows no sign of the disease, but his brother has epilepsy and enuresis, which suggests the possibility of syphilis. Another brother has an interstitial keratitis which makes us take a blood for a Wassermann Reaction, then the light breaks upon us.

"Of course we should make a Wassermann upon the mother of a child showing active signs or symptoms of syphilis, and a positive reaction will often lead us aright. But here we encounter the curious fact that the mother of a luetic child may give a negative Wassermann Reaction, because while carrying her syphilitic fetus, she has attained an immunity without, as was at one time thought, acquiring the disease herself. Such mothers are those referred to by Colle, in promulgating his well-known Law, to-wit, that a woman giving berth to a luetic child and showing symptoms of the disease herself cannot be infected by her child. She is immune to syphilis but not syphilitic. Recent observation, however, has shown that if these mothers are examined directly after the birth of their syphilitic offspring nearly one hundred per cent of them will show a positive reaction. We therefore now believe that when a negative reaction does occur in such mothers there has been a spontaneous cure, but that they were at one time truly syphilitic.

"Some of the stigmata of congenital lues which should always make us suspicious are idiocy, imbecility, visceral, bone and skin manifestations, fatigueability and excitability at puberty, headache, especially at night, a moody temperament and a lack of joy and interest in things for one so young, snuffles, saddle nose, Hutchinson's teeth, corneal opacities, defective hearing, scaphoid scapula, bulging of the frontal eminences, enlarged epitrochlear glands, choroiditis, optic neuritis, irregularity in the shape of the pupils, enlarged spleen and liver not accounted for otherwise, and delayed closure of the fontanelles.

"In tertiary and congenital syphilis, the socalled late hereditary syphilis, the Luetin test has been of great value in my hands in indicating the probable cause of many obscure conditions and is, in my judgment, well worth consideration by those of us who are engaged in the general practice of Medicine."

Dr. H. R. Donaldson emphasized the importance of making the Wassermann a routine procedure in all suspicious cases. He thinks that it is ignorance on the part of the father which causes so many congenital syphilitics. He reported three cases where he has delivered stillborn children and where he has later, after appropriate treatment, delivered healthy, well developed children. In closing Dr. Donaldson again stressed the value of the Wassermann as a routine measure.

In discussing Dr. Lindorm's paper Dr. C. C. Aven mentioned the close relationship existing between syphilis and tuberculosis. He stated that when a man knows syphilis and tuberculosis in all their various manifestations, that such a man is well versed in Medicine. Syphilis of the lungs is one phase of the disease which should be more carefully looked for and studied.

Dr. R. R. Daly mentioned the difference between inherited and congenital syphilis. He said that it is impossible for the father to transmit syphilis directly to the child but that the mother must become infected in all these cases. When syphilis is looked for more carefully and eliminated more thoroughly the race will be greatly benefited.

Dr. E. C. Thrash said that the terms congenital and inherited syphilis were used interchangeably, but that it is impossible for the child to inherit syphilis from a biological standpoint. The father (of the case reported by Dr. Lindorme) could have given syphilis to the child by kissing it or other direct means, but it may have become infected in utero. Dr. Thrash said that there are syphilitic carriers just as there are carriers of all other chronic diseases. He differentiates an academic from a clinical type of syphilis. In the academic type there are no external or visible manifestations of the disease and the patient is in an apparently normal condition. However, this patient may give the disease to another who may have a very severe type of the disease. The reason the father could not give syphilis to the child directly is because if the spermatozoon contained a spirochete it would be almost impossible for it to fertilize an ovum and if it did fertilize an ovum it would kill it.

Dr. Richard M. Nelson called attention to the fact that it has been shown recently that after taking iodides a patient will give a positive reaction to the Luetin Test.

In closing Dr. Londorme, in reply to a question from Dr. Selman, said that he would try to get a Wassermann made on the father and mother of the case reported by him.

Dr. H. R. Donaldson reported a case in which he had done a nephrectomy five years ago in which he tied the renal vessels and ureter with braided silk. The patient made a rapid and uneventful recovery. About four months ago the patient came to him complaining of pain in the side from which the kidney was removed and also in the bladder. The patient passed in his presence per urethra the silk ligature which he tied around the ureter five years ago. Three days later the patient passed a second ligature which he thinks is another which he put on at that time. Dr. Donaldson exhibited the ligatures for inspection.

